# Studying the Safety of Trophectoderm Biopsy Using Visible Femtosecond Laser Pulses

**DOI:** 10.3390/ijms27188059

**Published:** 2026-09-10

**Authors:** Dmitry S. Sitnikov, Maxim A. Filatov, Viktoria S. Agentova, Inna V. Il’ina, Maria A. Ovchinnikova, Mikhail A. Ovchinnikov, Marina V. Kubekina, Anna V. Tvorogova, Yuliya Yu. Silaeva

**Affiliations:** 1 Joint Institute for High Temperatures, Russian Academy of Sciences, Moscow 125412, Russia; maxfilat@yandex.ru (M.A.F.); agentova.vika@bk.ru (V.S.A.); ilyina_inna@mail.ru (I.V.I.); mashaovtch2013@gmail.com (M.A.O.); mikalovtch@gmail.com (M.A.O.); marykumy@gmail.com (M.V.K.); yulya.silaeva@gmail.com (Y.Y.S.); 2 Institute of Gene Biology, Russian Academy of Sciences, Moscow 119334, Russia; annatvor@mail.ru; 3 Institute for Regenerative Medicine, Sechenov University, Moscow 119991, Russia; 4 Core Facility Centre, Institute of Gene Biology, Russian Academy of Sciences, Moscow 119334, Russia

**Keywords:** mice, trophectoderm biopsy, femtosecond laser, reactive oxygen species, heat shock proteins, implantation rate

## Abstract

Laser-assisted trophectoderm (TE) biopsy is typically performed with millisecond diode lasers, where thermal effects remain a major safety concern. Femtosecond lasers offer a fundamentally different interaction regime, but their safety profile for TE biopsy has not been evaluated. This study aimed to assess the potential of a femtosecond laser as a tool for TE biopsy in CD1 mouse blastocysts. Biopsies were performed using single pulses at a wavelength of 514 nm with peak intensities of 5.3–16 TW/cm^2^. The number of pulses required to separate TE samples of different isthmus widths was quantified. The study of biological effects included reactive oxygen species (ROS) and heat-shock proteins (HSP70). Laser exposure at 16 TW/cm^2^ enabled an efficient TE biopsy within a clinically relevant number of pulses, without increasing ROS levels and with only limited HSP70 elevation, consistent with an adaptive stress response. A total of 417 embryos were transferred to pseudopregnant females to assess implantation and developmental outcomes. Implantation rates (25.8% vs. 29.6%) and the distribution of developmental outcomes (normal embryos, developmental delay and resorption) did not differ significantly between biopsied and non-exposed blastocysts in the control groups. This supports the further evaluation of femtosecond source as a tool for preimplantation genetic testing procedures.

## 1. Introduction

Preimplantation genetic testing (PGT) at the blastocyst stage with trophectoderm (TE) cell biopsy has become a standard approach to reduce implantation failure and spontaneous miscarriage in patients undergoing assisted reproductive technology (ART) programs. The transition from blastomere biopsy to TE biopsy made it possible to increase the informativeness and reliability of the analysis [1,2], as well as to improve the safety of the procedure [3]. This has been reflected in increased pregnancy and live birth rates [4]. However, the biopsy procedure itself involves several technical steps (assisted hatching, cell aspiration and biopsy sample separation), each of which can potentially affect the embryo and subsequent perinatal outcomes. The first of these stages is assisted hatching, which is required when the natural breach of the *zona pellucida* (ZP) is hindered due to morphological or biochemical abnormalities caused by age-related factors, genetic predisposition or the cryopreservation procedure [5,6,7]. Assisted hatching can be performed using various methods, including mechanical instruments, chemical agents (acid Tyrode’s solution, pronase), and millisecond lasers [8]. Laser-assisted hatching (LAH) is considered less traumatic, less labor-intensive and faster than the other two methods [4].

A trophectoderm cell biopsy can be performed in several ways, using lasers, mechanical instruments, or a combination of both. The development of the latter continues, as evidenced by the creation of improved microneedles and pipettes that facilitate the embryologist’s task [9,10]. Similar to laser-assisted hatching, laser biopsy is currently performed in modern clinics using pulsed semiconductor laser dissectors with typical parameters (pulse duration of 0.6 ms or more, power of ~250 mW, spot diameter of about 3 µm, and radiation wavelength of 1480 nm). Although laser-assisted trophectoderm biopsy is considered a safer and more controlled approach than mechanical methods, largely due to standardized laser hatching protocols and shorter manipulation time, data from direct comparative studies do not confirm its superiority in terms of embryo survival and clinical outcomes [4,8,11]. The main concerns regarding laser-assisted biopsy are the thermal effects it may have and its potential influence on the incidence of mosaicism in the embryo [12].

The underlying reason lies in the physics of laser exposure itself. Studies have shown that, under certain conditions, the temperature at the center of the spot can reach several hundred degrees and the heated region can extend beyond the laser spot’s geometric dimensions [13,14]. For this reason, trophectoderm cell biopsy is considered the most critical and potentially traumatic stage of an ART cycle. It requires experienced specialists to perform the procedure in strict compliance with approved protocols.

Due to their extremely short pulse duration, high peak power, and predominantly photodisruptive interaction mechanism with minimal lateral heat spread from the exposure region, femtosecond lasers are considered a promising alternative to millisecond systems [15]. Consequently, it becomes possible to form thin incisions in the ZP that are comparable in width to the laser spot size of about a micrometer in diameter [16], and to even perform embryo labeling [17]. Previously, these systems were used for LAH [18,19]. The aforementioned features of femtosecond laser pulses theoretically allow for a reduction in collateral damage during biopsy sample separation. However, substantially fewer studies have examined the use of femtosecond pulses for TE biopsy compared to standard, clinically validated millisecond laser systems. In particular, few studies have evaluated microsurgical efficiency (i.e., the number of pulses required to separate a biopsy sample at a given width of isthmus formed outside the ZP) without examining the early cellular stress response and the long-term reproductive outcomes [20,21].

It is known that exposing a cell to a laser can lead to transient changes in the level of reactive oxygen species (ROS) and the induction of heat-shock proteins. These are early markers of cellular stress that may precede structural damage. However, clinical studies typically focus on pregnancy rates, implantation, and neonatal complications [22,23], which only indirectly reflect the degree of cellular stress induced by a particular biopsy protocol. Therefore, using a standardized mouse blastocyst model that allows one to vary the intensity of femtosecond radiation, control the number of pulses, and quantitatively assess ROS and HSP70 expression is an important step toward substantiating the safety of such methods.

The present study extends previous feasibility studies [20,21] in three respects: (i) it aimed to quantitatively characterize the relationship between the geometry of the isthmus formed during hatching and the number of femtosecond pulses in the visible spectral range required to separate the TE biopsy sample in a mouse blastocyst model; (ii) the study also assessed the early cellular stress response (ROS level and HSP70 expression) after biopsy at different laser radiation intensities and the implantation potential of embryos; (iii) and it related the physical parameters of femtosecond laser dissection to molecular stress markers and clinically relevant implantation outcomes. Thus, the novelty of the present work is the integrated evaluation of microsurgical efficiency and biological safety of visible femtosecond TE biopsy rather than the demonstration of the biopsy technique alone. It creates a basis for translating optimized femtosecond TE biopsy modes into clinical preimplantation genetic testing practice in the future.

## 2. Results

This section first outlines the femtosecond biopsy workflow, comprising laser-assisted hatching and subsequent trophectoderm cell separation. The study design required identifying the optimal laser exposure parameters for TE biopsy. Accordingly, data on biopsy efficiency are presented across a range of laser intensities and diverse isthmus geometries. Once the most effective exposure conditions were identified, the biological effects were examined and the early cellular stress response was evaluated using analyses of reactive oxygen species and heat-shock proteins. In the final stage of the study, implantation and developmental outcomes after transfer of biopsied embryos were evaluated to determine the safety of the procedure at the whole-organism level.

### 2.1. Laser-Assisted Hatching

**Figure 1:**
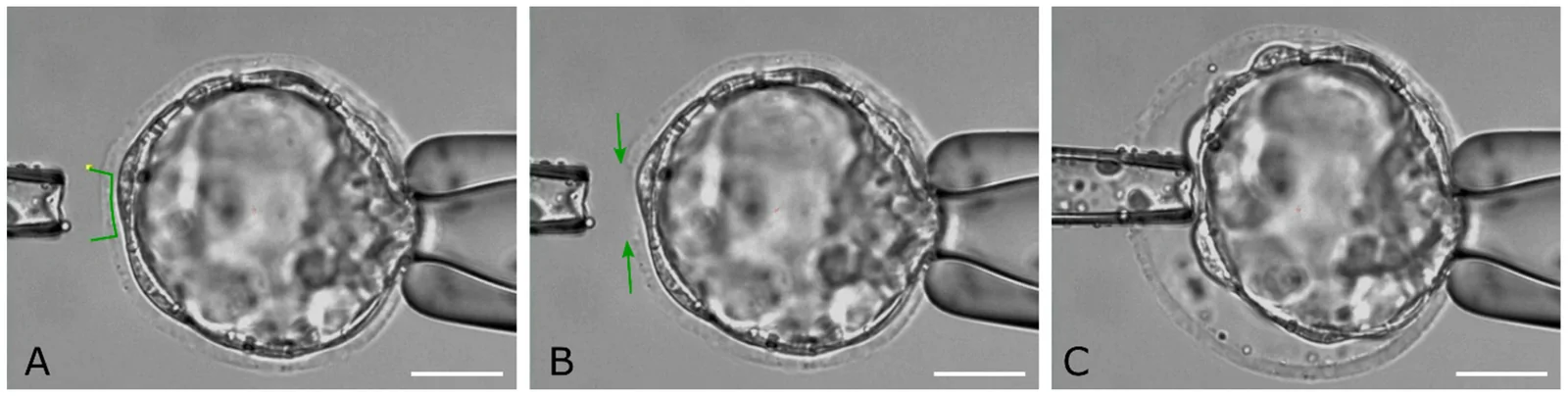
Laser-assisted hatching using femtosecond laser pulses (**A**) prior to LAH and (**B**) post-LAH. Green polyline and arrows indicate the laser beam’s trajectory and the formed ZP thinning. (**C**) Ready for suctioning of TE cells. The scale bar is 30 µm.

When LAH was required, the embryo was held in place by a holding pipette using the ZP on the side of the inner cell mass (ICM). An example of the applied trajectory for subsequent laser dissection and the result of microsurgery of the embryo *zona pellucida* are presented in Figure 1A,B. After thinning the shell, the operator could enter the blastocyst ZP with the pipette to aspirate TE cells (Figure 1C).

### 2.2. TE Cell Biopsy

**Figure 2:**
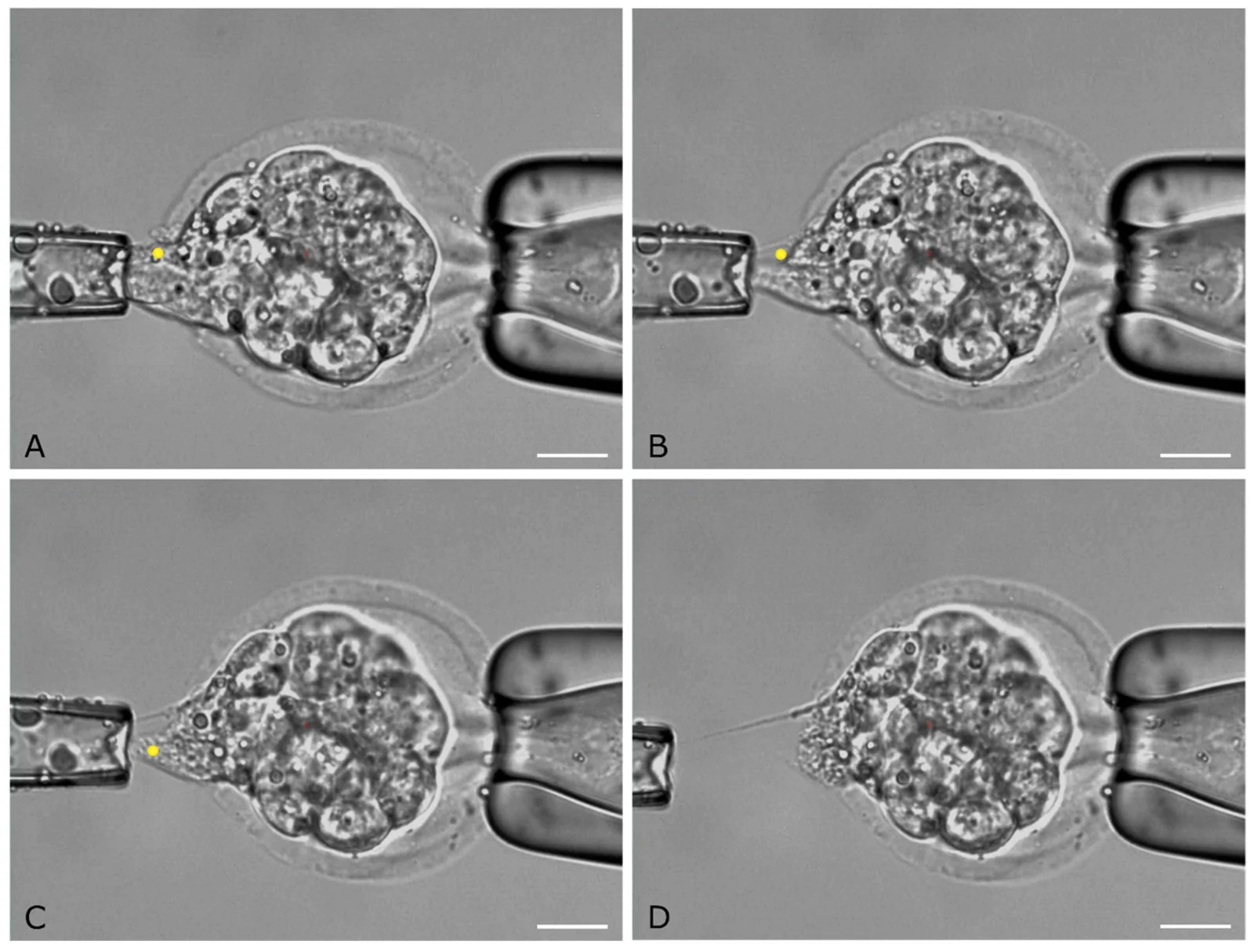
Trophectoderm cell biopsy of a blastocyst using femtosecond laser pulses: (**A**) cell aspiration, (**B**) after the first exposure; (**C**) before and (**D**) after the second exposure. The yellow marker indicates the exposure site. The scale bar is 20 µm.

After capturing the TE cells with the biopsy pipette and creating tension (Figure 2A), the operator selected a site and performed laser exposure. The distance from the edge of the isthmus was determined empirically for each intensity and did not exceed 8 μm in the experiment. After partial dissection (Figure 2B), the exposure site was shifted farther from the edge (Figure 2C). This procedure was repeated until the entire width was cut. The number of laser pulses needed to detach the biopsy sample depended on the exposure parameters of the laser and the width of the TE cell isthmus to be separated.

Laser exposure during the procedure lasted from a few seconds up to several tens of seconds, depending on the number of pulses required for a given isthmus width. Throughout this interval, no membrane blebbing or localized darkening/opacification of the cytoplasm—both expected visual correlates of acute cytotoxic or thermal injury—was observed in TE cells adjacent to the exposure site. The boundaries of neighboring cells remained sharply defined even for the highest pulse intensities.

During the separation of the biopsy sample, the blastocyst commonly collapsed into a compact cell mass, which precluded assessment of cytoplasmic vacuolization at this stage. However, within a few hours, the blastocyst expanded back to its normal shape. Confocal microscopy examination of the re-expanded embryos at this later time point revealed no visual anomalies. Quantitative assessment of cell viability by propidium iodide (PI) staining (see Section 2.4) showed no evidence of widespread cell death associated with the biopsy procedure.

### 2.3. Assessment of TE Cell Biopsy Efficiency at Different Femtosecond Pulse Intensities

**Figure 3:**
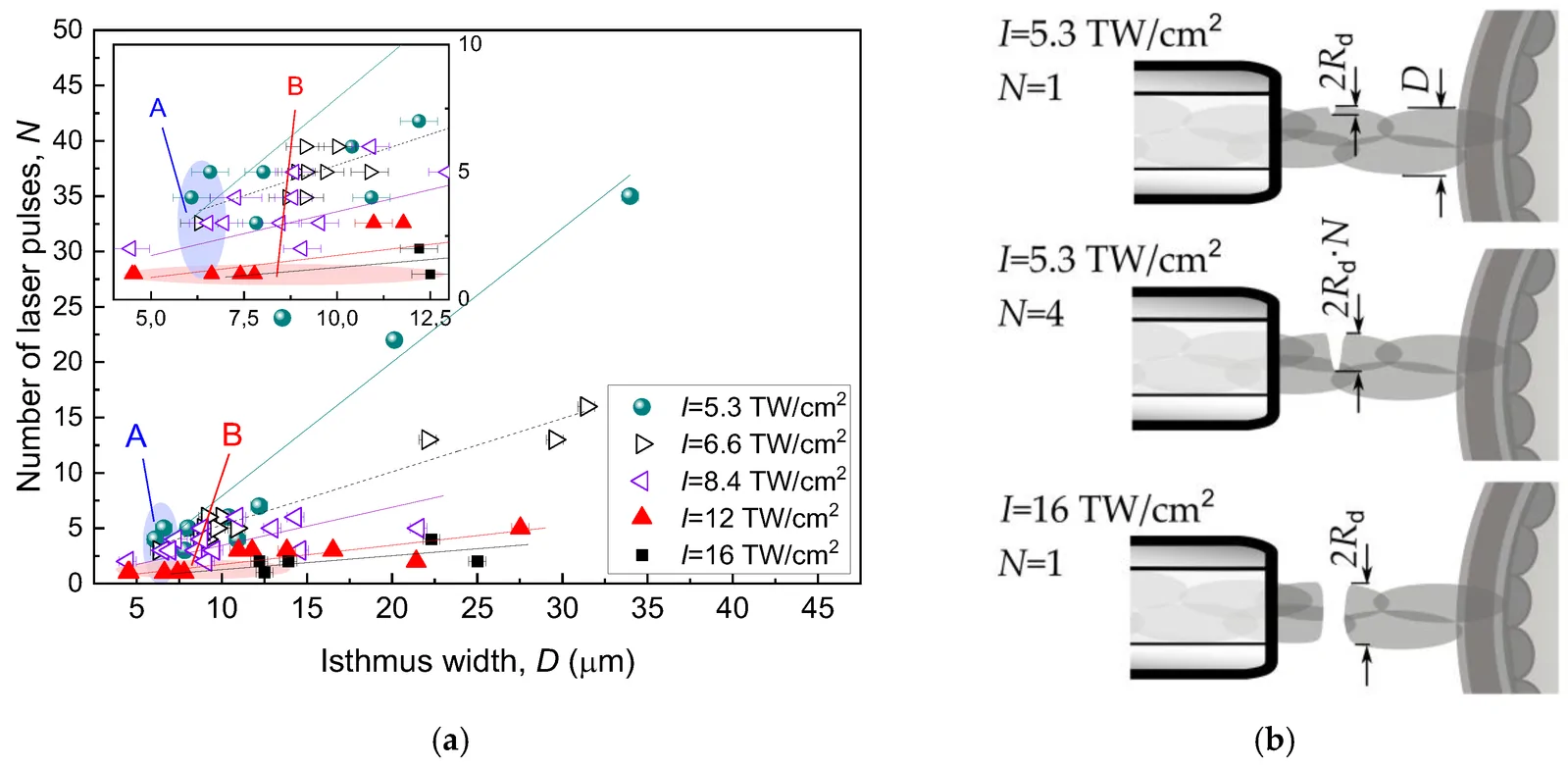
(**a**) Dependence of the number of pulses *N* required for biopsy sample separation on the isthmus width *D* at different pulse intensities *I*. (**b**) Schematic comparison of TE cell isthmus separation at low (5.3 TW/cm^2^) and high (16 TW/cm^2^) pulse intensities; *D* = 15 µm; *R*_d_—disruption radius.

All intensities reported in this section refer to peak intensity at the focal region; the laser-beam focal spot radius was approximately 1.4 µm at the 1/*e* level (see Section 4.9 for details). The results of experiments on separating biopsy samples with different isthmus widths *D* using single laser pulses at intensity *I* = 5.3–16 TW/cm^2^ are presented in Figure 3.

An intensity of 5.3 TW/cm^2^ proved to be close to the threshold value at which trophectoderm biopsies using visible femtosecond pulses are possible. A distinctive feature of these pulses is the delicate laser exposure they provide. The size of the cavitation bubble formed due to optical breakdown in the cytoplasm is small (approximately 1 µm), as is the size of the pores formed in the cell membrane. It is believed that their diameters do not exceed the bubble size [24]. At *I* = 5.3 TW/cm^2^, careful selection of the exposure site was required, both in the horizontal plane and along the laser beam axis to achieve membrane dissection. The distance from the isthmus edge (let us denote it as 2*R*_d_) was typically about 1 µm. It should be noted that 2*R*_d_ was chosen empirically for each intensity, starting from about 1 µm for 5.3 TW/cm^2^ and ending with approximately 8 µm for 16 TW/cm^2^. Too large a distance from the edge resulted in cutting the center of the cell while leaving the edge uncut (see a thin “thread” in Figure 2B–D left after the first laser exposure). Such unwanted threads required additional time or laser shots to detach.

The hatching begins with the formation of a rupture in the *zona pellucida* of the embryo and the emergence of a single cell, followed by several more cells, beyond the zona. The typical width *D* of such a narrow isthmus during hatching was approximately 6 ± 1 µm (except for a few cases with *D* = 3 µm)—this is marked as region “A” in Figure 3a. At least several laser pulses with an intensity of 5.3 TW/cm^2^ (*N* = 4–6) were required to separate a biopsy sample even at such a small isthmus width. A higher *D* required a larger *N*, which reached several tens in the experiment. Meanwhile, the procedure became more time-consuming and laborious. For this reason, the laser pulse intensity of 5.3 TW/cm^2^ could hardly be considered optimal for the biopsy task.

Increasing *I* made it possible to reduce the required number of pulses *N* for any value of *D*. The isthmus of minimal width (region “A” in Figure 3a) could be separated in two to three pulses at an intensity of 8 TW/cm^2^ and in a single pulse at 16 TW/cm^2^. Laser biopsy with pulses of maximum intensity was considered the most efficient method and enabled the separation of samples with an isthmus width of up to 12–15 µm (region “B” in Figure 3a) in a single pulse. However, in addition to small cavitation bubbles, large vapor-gas bubbles formed with radii of up to ten micrometers. Higher intensities were not considered.

To formalize the differences in the *N*(*D*) dependencies for various *I* values shown in Figure 3a, a linear model with an interaction term between *D* and *I* was used, along with a multivariate test of the significance of the interaction coefficients (Wald test). The *D* × *I* interaction was found to be statistically significant (*p* ≈ 3.4 × 10^−6^), indicating differences in the slopes of *N*(*D*) at different intensities. This result confirms the observed change in biopsy efficiency; pulses with intensities of 12 and 16 TW/cm^2^ appear to be preferable for cell separation. Isthmuses of width *D* ~ 20 µm can be cut in 3 ± 1 pulses, and careful aiming along the optical axis is unnecessary, unlike with intensities close to the threshold of 5.3 TW/cm^2^.

### 2.4. Assessment of ROS Level in Embryo Cells After TE Cell Biopsy

Embryos that had begun hatching were used in the experiment. To assess the effects of laser exposure, intensities of 12 and 16 TW/cm^2^ were chosen because they make it possible to separate the biopsy sample with a small number of laser pulses. These two specific intensities were chosen rather than lower values to avoid confounding the effect of peak intensity with differences in pulse number. At near-threshold *I*, substantially more pulses are required to separate an isthmus of comparable width. Therefore, any biological effect would reflect both intensity and cumulative pulse exposure rather than intensity alone. This consideration guided the selection of isthmus widths and pulse numbers.

**Figure 4:**
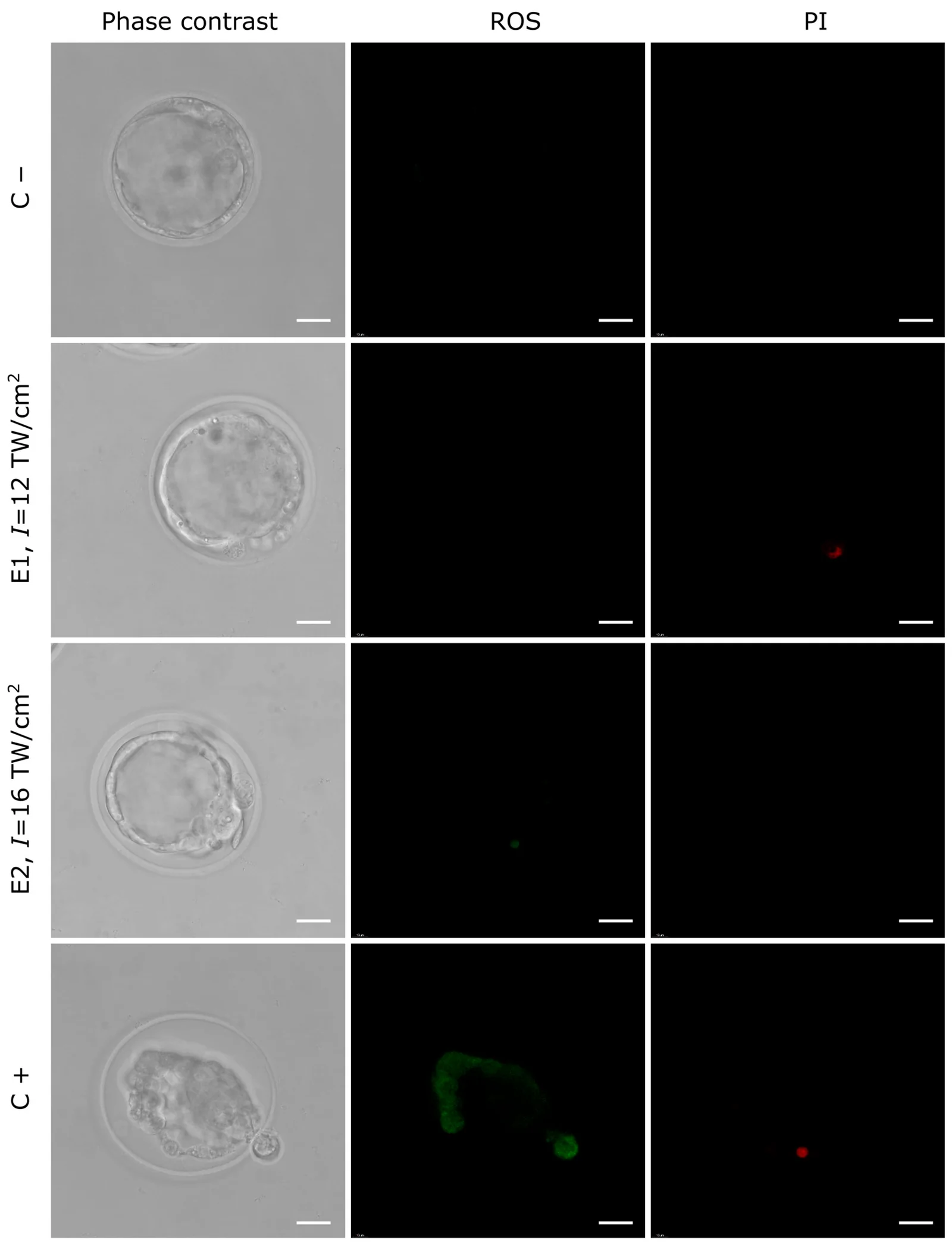
Results of ROS analysis in embryos. The number of laser pulses in the experimental groups is *N* = 2. Pseudocolors: ROS—green; PI—red. Scale bar is 20 µm.

Two experimental groups (E1 and E2) and two control groups (negative and positive) of five embryos each were formed. Comparisons were performed between each experimental group and the control groups, as well as between groups E1 and E2. Embryos with thin isthmuses (*D* ~ 5–8 µm), which could be separated with two laser pulses, were selected for these groups. The confocal microscopy results for all groups are presented in Figure 4. Propidium iodide (PI), which stains dead cells, is shown in red, and reactive oxygen species are shown in green.

Notably, brightly stained individual cells were not observed adjacent to the cells of the biopsy sample undergoing separation. The cells of the embryos in the experimental groups demonstrated weak green fluorescence, comparable to the negative control. Only one embryo in the E2 group (*I* = 16 TW/cm^2^) showed an exception in the form of a single fluorescent cell with an increased ROS level, as shown in Figure 4. One exception, a cell stained positively with propidium iodide, was found in group E1 (*I* = 12 TW/cm^2^) and is also presented in the figure.

**Figure 5:**
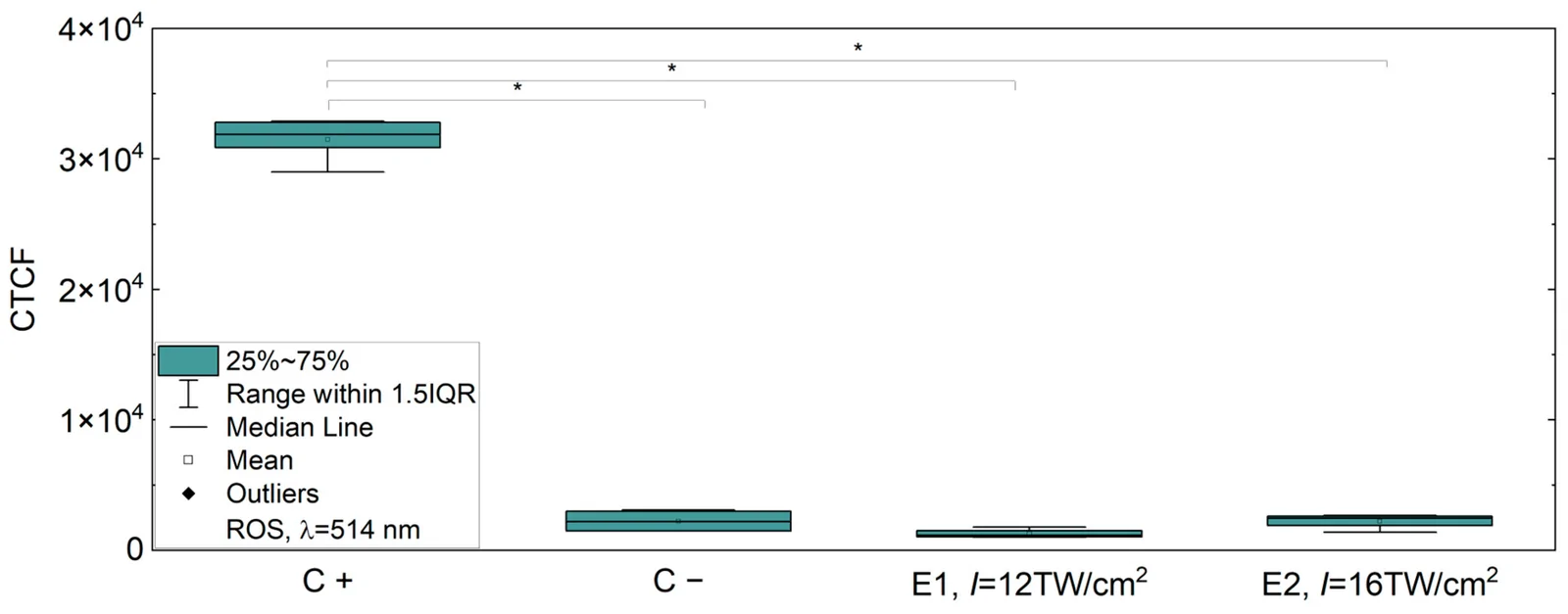
Relative ROS levels in four embryo groups: positive and negative control and experimental groups E1 and E2 at *I* = 12 TW/cm^2^ and *I* = 16 TW/cm^2^. The symbol (*) denotes statistically significant differences between groups according to the Mann–Whitney U test with Bonferroni correction (*p* < 0.05).

A quantitative assessment of the results was performed using the ImageJ cell fluorescence measurement method for an image containing cumulative embryo cell brightness information across all confocal image layers [25]. The corrected total cell fluorescence (CTCF) was calculated. The Mann–Whitney U test with Bonferroni correction was used to assess the statistical significance of differences in brightness levels between groups because the distributions deviated from normality (Shapiro–Wilk test). The results of the statistical analysis are presented in Figure 5.

A quantitative assessment of ROS levels showed that, after biopsy, there were no statistically significant differences between the experimental groups (E1, *I* = 12 TW/cm^2^; E2, *I* = 16 TW/cm^2^) and the negative control, “C−” (*p* values are presented in Table 1). To increase informativeness, effect sizes were also calculated. The Hodges–Lehmann estimate for the difference between E1 and “C−” was −1085 (95% CI: −1815 to +327); for the difference between E2 and “C−”, it was −101 (95% CI: −777 to +1036); and for the difference between E2 and E1, it was +1091 (95% CI: +330 to +1532). The corresponding Cliff’s delta values were −0.68 (95% CI: −1 to −0.04), −0.1 (95% CI: −0.8 to +0.6), and 0.8 (95% CI: 0.2 to 1.0). As expected, the positive control “C+” demonstrated a significant increase in signal compared to the negative control “C−” (Hodges–Lehmann estimate: +29,640; 95% CI: +27,519 to +30,640; Cliff’s delta: 1.00). The results indicate that laser pulses with the maximum intensity used in this experiment can be used for trophectoderm cell biopsy, as laser exposure does not increase ROS levels.

### 2.5. Assessment of Heat-Shock Protein Expression Level in Embryo Cells After TE Cell Biopsy

**Figure 6:**
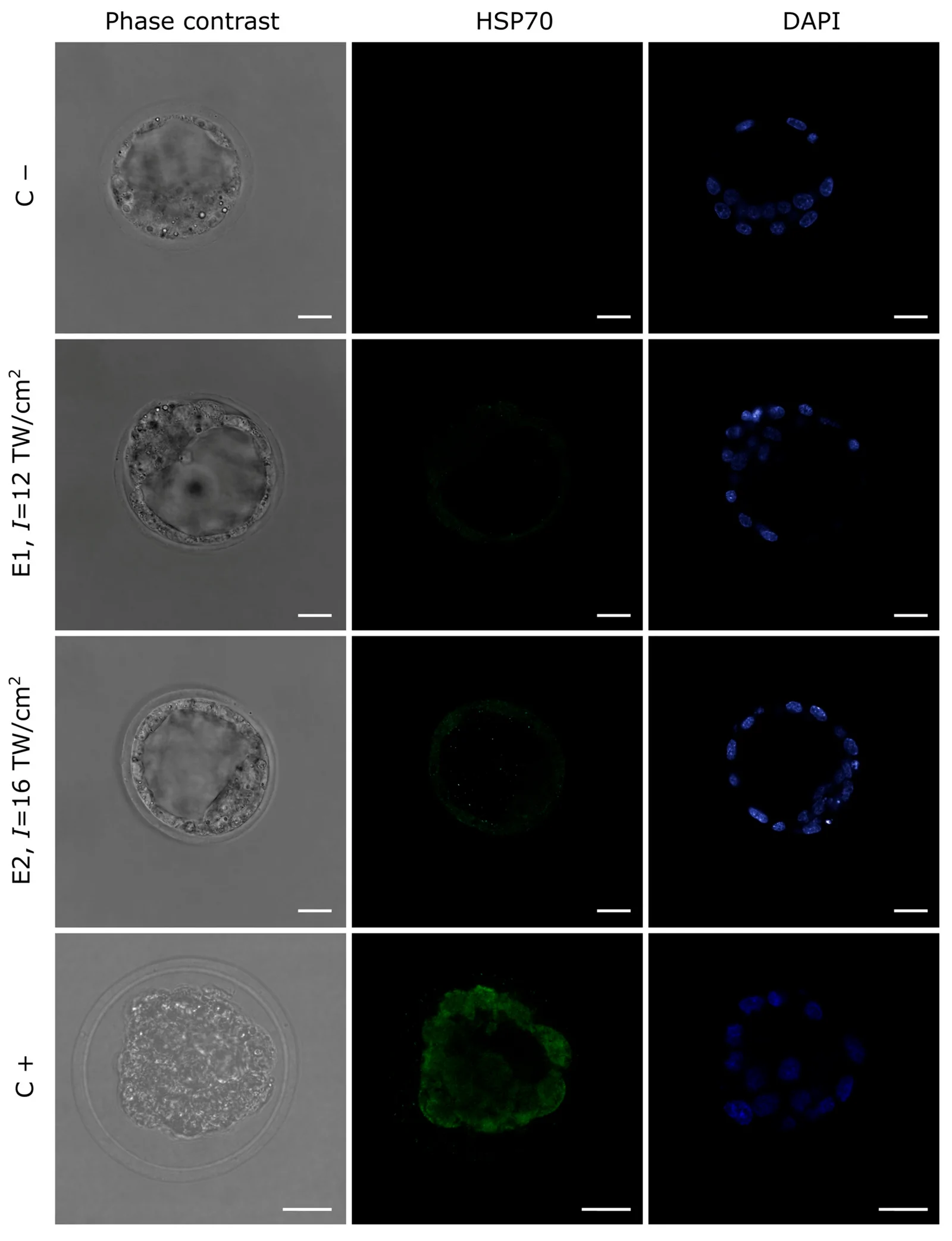
Results of *Hsp70* production analysis in stained embryos. The number of laser pulses in the experimental groups is *N* = 2. Pseudocolors: HSP—green; DAPI—blue. Scale bar is 20 µm.

To determine the levels of heat-shock protein HSP70 expression in the cells of the embryo, an analysis of images of blastocysts (using the confocal microscopy method) stained with antibodies against this protein was performed (see Figure 6). Following the ROS experiment, two groups of five embryos each were formed: E1 (*I* = 12 TW/cm^2^) and E2 (*I* = 16 TW/cm^2^). Negative and positive control groups included ten and five embryos respectively. The same two intensities, and the same rationale for their selection (see Section 2.4), were applied to the HSP70 assessment. Isthmuses of comparable width (*D* ≈ 5–8 µm) were separated with the same number of pulses (*N* = 2) at both intensities, allowing the effect of peak intensity on HSP70 expression to be evaluated independently of pulse number.

**Figure 7:**
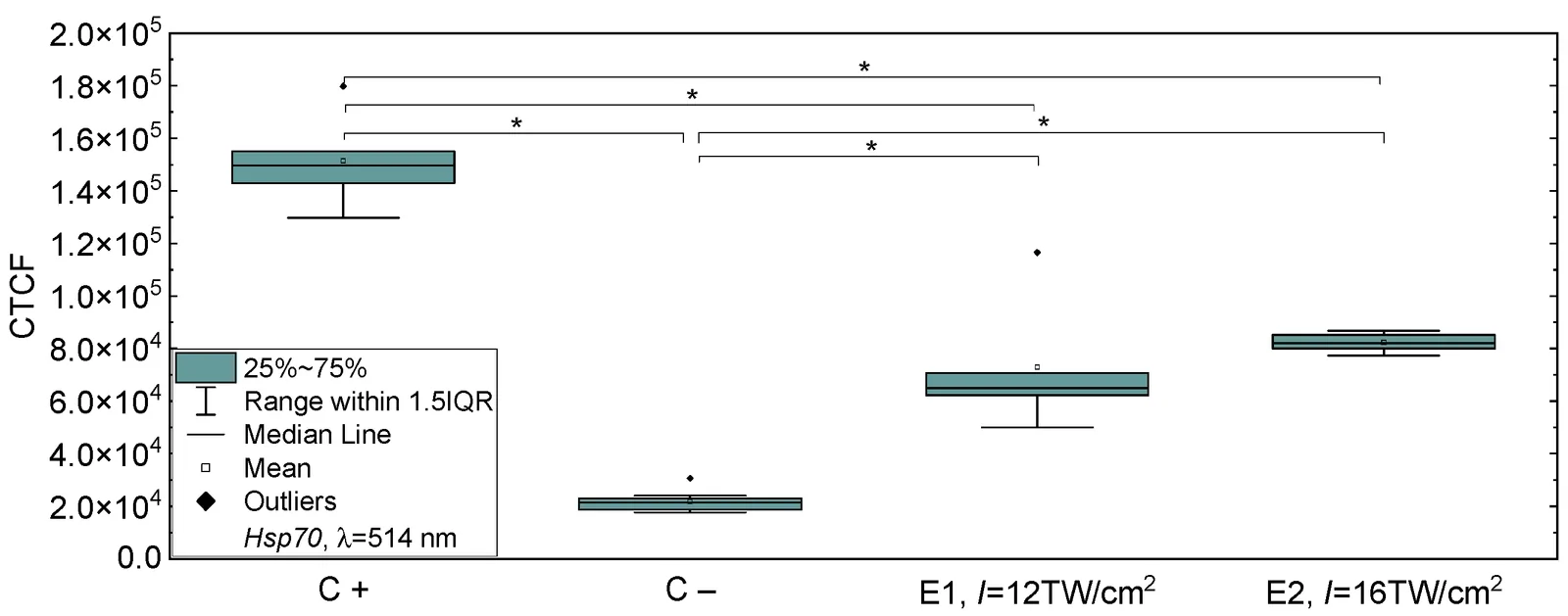
Relative levels of *Hsp70* protein in four embryo groups: positive and negative control and experimental groups E1 and E2 at *I* = 12 TW/cm^2^ and *I* = 16 TW/cm^2^. The symbol (*) denotes statistically significant differences between groups according to the Mann–Whitney U test with Bonferroni correction (*p* < 0.05).

Analysis of HSP70 fluorescence intensity (Figure 7) revealed increased heat-shock protein production in the experimental groups compared with the negative control “C−”, with a stronger response observed at higher exposure intensity (E2 compared with E1). The positive control demonstrated the highest values (Mann–Whitney U test with Bonferroni correction; *p* values are given in Table 2). In terms of effect sizes, the Hodges–Lehmann estimate for the difference between E1 and “C−” was +44,012 (95% CI: +31,271 to +92,545); for the difference between E2 and “C−” it was +61,206 (95% CI: +57,033 to +64,540); and for the difference between E2 and E1 it was +16,191 (95% CI: −29,738 to +27,229). Cliff’s delta values for the comparisons E1 and “C−” and E2 and “C−” were 1.00; for the comparison E2 and E1 it was 0.60 (95% CI: −0.2 to +1.0). The positive control “C+” substantially exceeded “C−” (Hodges–Lehmann estimate: +127,354; 95% CI: +111,045 to +155,811; Cliff’s delta: 1.00).

Taken together, the results of the biological-effects analysis indicate that exposure to femtosecond laser pulses with an intensity of *I* = 12 TW/cm^2^ at a wavelength of λ = 514 nm does not lead to an increase in reactive oxygen species production in embryo cells; however, it does promote some growth in heat-shock protein (HSP70) production. As laser pulse intensity increases, heat-shock protein production also rises. This suggests that further increases in intensity may lead to enhanced HSP production and negatively affect the condition of embryo cells.

### 2.6. Study of Embryo Implantation After TE Biopsy

**Figure 8:**
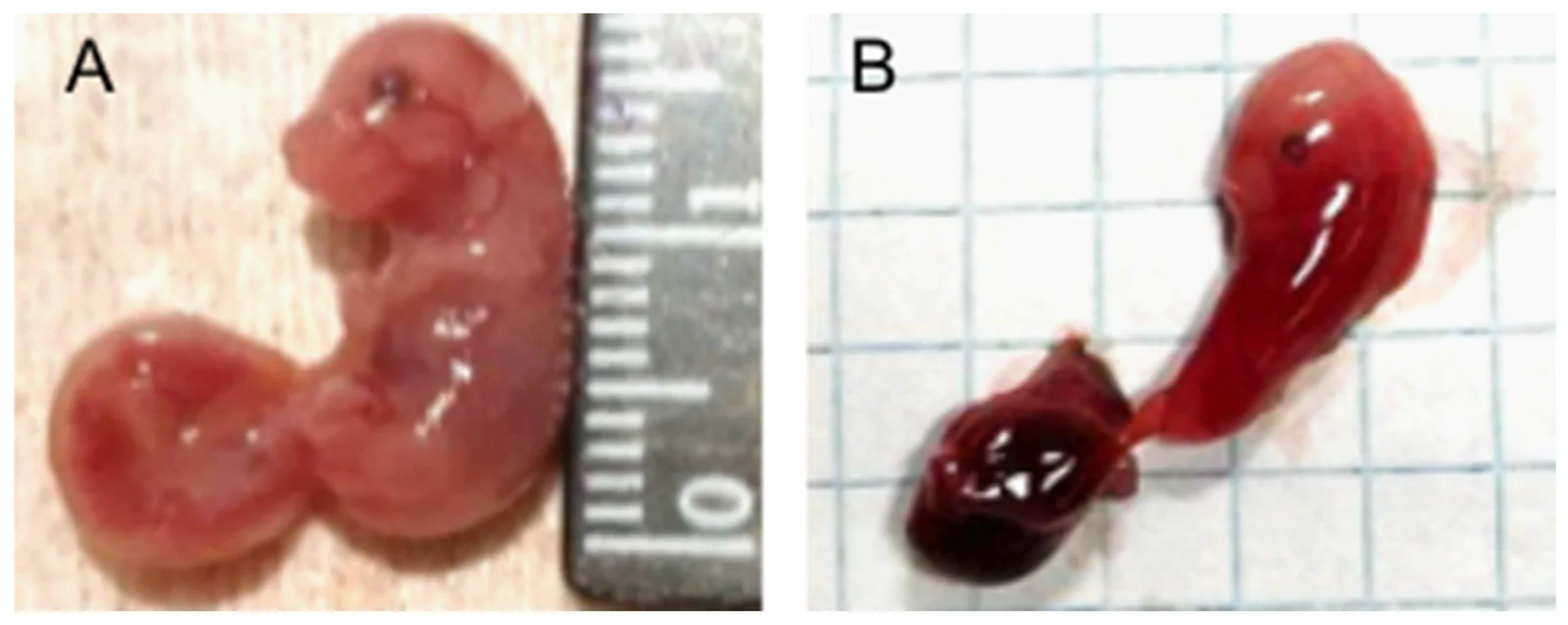
Embryos on day 19 of development (E18.5): (**A**) without pathology and (**B**) with developmental delay.

In order to investigate the safety of TE cell biopsy using femtosecond laser pulses, the maximum intensity of 16 TW/cm^2^ was selected as the worst-case scenario. Embryos obtained after TE biopsy, as well as those from the control group, were transferred to pseudopregnant females. At stages E16.5–E18.5, the surrogate mothers were euthanized and examined anatomically to analyze the condition of the embryos and any resorptions. Typically, a mouse embryo with no pathological conditions on embryonic development days 18.5–20.5 (E18.5–E20.5) measures up to 2 cm in length and 1.5 cm in width. The amniotic membrane is intact, the placenta is plethoric and a heartbeat is observed. Upon opening the amnion sac, formed limbs, a tail, ears and eyeballs can be detected (Figure 8A). Upon contact, a response to stimulation is observed. In contrast, an embryo with developmental delay is smaller in size, with underdeveloped limbs and tail, and an absent heartbeat (Figure 8B). In cases of abnormal development, individual embryos may undergo resorption. A large number of resorptions indicates the presence of negative factors. However, this phenomenon was observed in both the experimental and control groups, including embryos that were not subjected to laser exposure (see Table 3).

The number of implantations *N*_i_ and the implantation rate *K*_ir_ = *N*_i_/*N*_t_ are given in Table 3, where *N*_t_ is the number of transferred embryos in each group. “E” denotes embryos without developmental pathology, “UE” denotes underdeveloped embryos and “R” denotes resorptions.

The implantation rate (summed across categories E + UE + R) was 25.79% (57/221) in the experimental group and 29.59% (58/196) in the control group. This corresponds to a risk difference of −3.80 percentage points (95% CI: −12.41 to +4.81). The relative risk of implantation was RR = 0.87 (95% CI: 0.64 to 1.18) and the odds ratio was OR = 0.83 (95% CI: 0.54 to 1.27). These results indicate an absence of statistically significant worsening of implantation in the experimental group. The results of the chi-squared test also did not reveal a statistically significant association between group membership (experimental vs. control) and the distribution of implantation outcomes (E, UE, and R). However, due to small expected frequencies (less than five) for the outcome “UE”, this result should be interpreted with caution.

Analysis of individual outcomes, calculated as a proportion of transferred embryos, showed that the rate of normal embryos “E” was 11.76% (26/221) in the experimental group and 15.31% (30/196) in the control group (risk difference: −3.54 percentage points; 95% CI: −10.13 to +3.05). Likewise, the rates of underdeveloped embryos “UE” and resorptions “R” did not show convincing differences between groups. For “UE”, the risk difference was −0.17 percentage points (95% CI: −2.47 to +2.12); for “R”, it was −0.08 percentage points (95% CI: −6.49 to +6.32). Among the implanted embryos, the proportion of outcomes “E” was 45.61% (26 out of 57) in the experimental group and 51.72% (30 out of 58) in the control group (a difference in proportions of −6.11 percentage points; 95% CI: −24.35 to +12.13). This also does not reveal a statistically significant shift in the structure of the outcomes.

### 2.7. Assessment of Biopsy Efficiency at Pulse Intensity I = 16 TW/cm^2^

**Figure 9:**
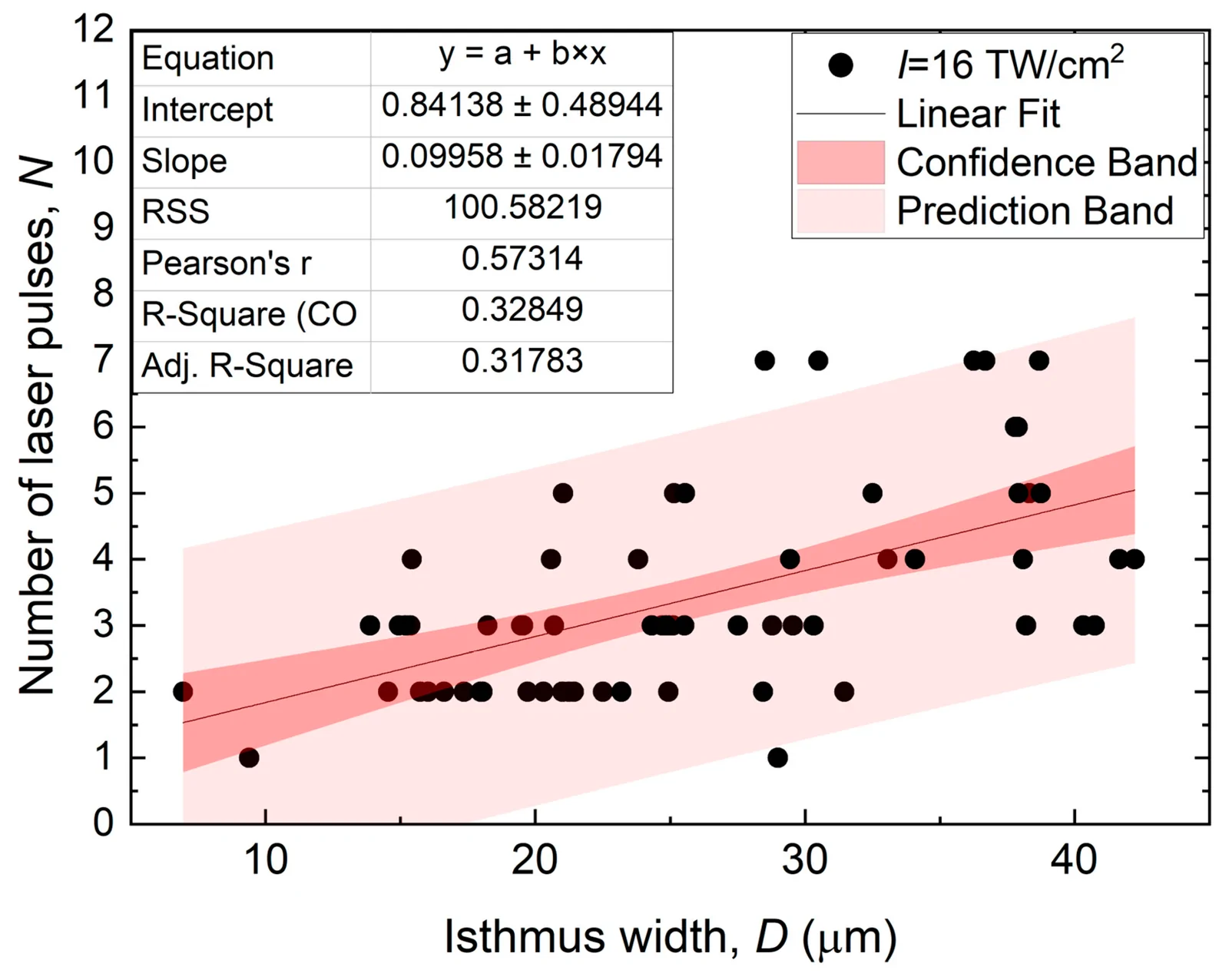
Dependence of the number of laser pulses *N* required for TE biopsy on the isthmus width *D*.

The experiment investigating the effect of TE biopsy using femtosecond pulses on embryo implantation enabled the collection of an extensive dataset on the relationship between the number of laser pulses *N* required to separate the biopsy sample and its width *D* (Figure 9). Linear regression revealed a statistically significant positive relationship between *D* and *N*. The fitted model was *N* = a*D* + b, where the slope was a = 0.0996 (95% CI: 0.0637 to 0.1354) and the y-intercept was b = 0.8414 (95% CI: −0.137 to 1.819). In physical terms, this corresponds to one pulse required for a biopsy of the thinnest isthmus, followed by an increase of approximately one pulse for every 10 µm increase in isthmus width (*t* = 5.55; *p* = 6.0 × 10^−7^). However, the model intercept is statistically compatible with zero (its 95% CI is −0.137 to 1.819), formally allowing for models with and without an intercept; the intercept model was retained for all reported predictions.

The model explained approximately one-third of the variability in *N* (*R*^2^ = 0.33, adjusted *R*^2^ = 0.32; Pearson’s *r* = 0.57), indicating a moderate linear association with considerable residual scatter. Residual analysis showed an approximately normal distribution (μ ≈ 0, σ ≈ 1.25) without pronounced systematic trends. On the basis of the obtained model, a 95% confidence band for the mean value and a 95% prediction band were constructed. The bands reflect, respectively, the uncertainty of estimation of the mean relationship and the comparatively wide range of individual observations at a given *D*. At *D* = 20 µm, the expected mean number of pulses was *N* ≈ 3.4 (95% CI ≈ 2.2 to 4.6). The 95% prediction interval covered approximately the range from one to five pulses, which reflects substantial variability even at fixed isthmus width.

Such factors as pre-tension of the biopsy sample created by the suction pipette, instability of pulse-to-pulse laser radiation energy, and the maturity grade and quality of the blastocyst may significantly contribute to the variability of the data. TE cells in grade 4 × B blastocysts (here × denotes any developmental stage of ICM) were considerably easier to detach (lower *N*) than those in grade 4 × A blastocysts. Pulling the biopsy sample facilitated cell separation, especially at large isthmus widths (*D* > 15 μm); this was probably due to elongation of the cells and reduction of their transverse dimension. However, controlling the value of the created pre-tension was not possible in the experiment.

## 3. Discussion

In this study, we applied second-harmonic radiation at a wavelength of 514 nm for TE biopsy for several reasons. This wavelength lies within a spectral window of comparatively low linear absorption in water-based biological media and is consistent with the parameters used in our earlier studies on femtosecond *zona pellucida* microsurgery and laser-assisted hatching. Because the diffraction-limited focal spot radius scales linearly with wavelength, the shorter 514 nm wavelength allows a smaller, more sharply defined focal volume than 1028 nm would provide under identical focusing conditions. This is advantageous for spatial precision. In addition, our own comparative experience with the same experimental platform suggests that near-infrared femtosecond exposure during LAH procedures required higher peak intensities than visible radiation at a wavelength of 514 nm. For instance, intensities of 6.5 TW/cm^2^ versus 2.5 TW/cm^2^ were required to create a 1.7 µm incision, consistent with the lower photon energy at 1028 nm [16,19].

A similar situation is observed in femtosecond TE biopsy: to reliably achieve optical breakdown and cell separation, peak intensities of approximately 10–65 TW/cm^2^ [20] are required for infrared pulses, whereas 5–16 TW/cm^2^ are sufficient for visible light. For these reasons, the 514 nm wavelength was selected as a practical engineering choice that provides efficient optical breakdown and precise microsurgery at moderate intensities.

The study evaluated two interrelated aspects of the use of femtosecond pulses for trophectoderm cell biopsy: the technological efficiency of separating the biopsy sample (through the *N*(*D*) relationship) and the biological safety of the procedure (through cellular stress markers and implantation results). The obtained *N*(*D*) data confirmed that isthmus width is a key geometric parameter that determines the required number of single laser pulses. However, the nature of this relationship changes when pulse intensity is altered. Values of 5.3, 6.6, 8.4, 12, and 16 TW/cm^2^ were considered.

Analysis of the *N*(*D*) data in a linear model with interaction (*N* ∼ *D* × *I*) identified a statistically significant *D* × *I* interaction (*p* ≈ 3.4 × 10^−6^), meaning the slopes of *N*(*D*) curves differ between intensity levels. In practical terms, increasing the intensity leads to a more “exposure-effective” biopsy procedure in terms of the number of laser pulses at a comparable *D*. This can be demonstrated visually and quantitatively. The linear model describes the expected mean number of pulses (non-integer *N*). After rounding the values to the nearest integer corresponding to the number of pulses actually used, the following is obtained: at *D* = 10 µm, the model predicts an average of about 8 pulses for *I* = 5.3 TW/cm^2^ and 1–2 pulses for *I* = 16 TW/cm^2^ (difference in mean values: 6.4 pulses; 95% CI: approximately from 3 to 10 pulses). At an isthmus width *D* = 20 µm, the expected mean *N* is approximately 20 pulses at 5.3 TW/cm^2^ and 2–3 pulses at 16 TW/cm^2^ (difference: 17.5; 95% CI: approximately from 11 to 24 pulses). From a practical point of view, such formalized confirmation justified the selection of 12–16 TW/cm^2^ as the most efficient modes. In these modes, *N* can be reduced at a comparable *D*, which shortens manipulation time and the total number of exposures. This reduces the time spent outside the incubator and decreases the labor intensity of the procedure.

This intensity-dependent efficiency can be rationalized in terms of the physical mechanism of femtosecond optical breakdown. Although the focal spot radius was only ~1.4 µm at the 1/*e* level (see Section 4.9), the laser-induced plasma generates a thermo-elastic stress (shock) wave whose mechanical action extends beyond the focal volume in three dimensions [26,27]. While the associated cavitation bubble remains sub-micrometric in radius near the breakdown threshold, the accompanying tensile stress wave propagates outward and decays gradually with distance. Membrane disruption occurs when the local tensile stress exceeds the membrane’s mechanical strength. This occurs whenever the breakdown epicenter lies within an effective disruption radius *R*_d_, regardless of whether the offset is lateral (distance from the isthmus edge) or axial (distance along the optical axis). Because *R*_d_ increases with pulse intensity, near-threshold exposures (*I* = 5.3 TW/cm^2^) required precise three-dimensional aiming, with the focus positioned within ~1 µm of the membrane in both the lateral plane and along the optical axis. At an intensity of 16 TW/cm^2^, *R*_d_ is substantially larger (empirically, up to ~8 µm), which simultaneously allows the exposure site to be positioned farther from the isthmus edge. This enables the separation of isthmuses up to 12–15 µm wide in a single pulse and relaxes the requirement for precise axial positioning since typical deviations of the isthmus surface from the focal plane fall well within *R*_d_. This unified picture accounts for both a reduced number of pulses and reduced precision requirements for aiming at higher intensities, including cutting a ~20 µm isthmus in 3 ± 1 pulses at *I* = 16 TW/cm^2^ without meticulous axial control.

From a physical standpoint, the key question is how the spatial precision and thermal effects of femtosecond pulses compare with those of the millisecond diode lasers currently used for trophectoderm biopsy. This comparison must take into account the fact that femtosecond systems operate in fundamentally different regimes. In our previous work on laser-assisted hatching [15], femtosecond pulses at a wavelength of 514 nm with a pulse duration of 280 fs and peak intensities up to 2.5–3 TW/cm^2^ were used below the threshold for optical breakdown. Under these conditions, nonlinear absorption occurs, resulting in plasma formation, which subsequently releases its energy to water. The numerical modeling applied was rather simple since it did not take into account phase transitions or the formation of cavitation bubbles. It was demonstrated that the maximum temperature increase at the center of the beam does not exceed approximately 2.5 °C. The temperature peak occurs around 10 ns after the pulse, and the heated region is confined to a micrometer-sized volume around the focal point.

In contrast, the current biopsy protocol operates at peak intensities of approximately 5–16 TW/cm^2^, clearly placing the interaction in the optical breakdown regime, as evidenced by the formation of vapor bubbles in the cytoplasm. Detailed experimental and theoretical studies of femtosecond laser breakdown in water [26,27] indicate that, at the breakdown threshold, the liquid irradiated within the focal volume is either smaller than or similar to the diffraction limit. This liquid is also superheated to temperatures on the order of 160–170 °C. At higher deposited energies, local temperatures can exceed 300 °C and trigger a phase explosion that drives the rapid formation of a cavitation bubble and a strong shock wave. A recent comprehensive energy-balance analysis [27] of laser-induced breakdown in water further suggests that the plasma region can briefly reach temperatures on the order of 10^3^ K and pressures in the gigapascal range. Meanwhile, a significant portion of the absorbed energy is converted into the mechanical work of bubble expansion and shock-wave emission rather than into long-term bulk heating.

The vapor generated during the breakdown largely condenses again during subsequent bubble oscillations. Consistent with this picture, the bubbles observed during trophectoderm biopsies in our experiments had diameters of approximately 1–2 µm at threshold intensity and up to 10–15 µm at higher intensities. The accompanying shock wave was sufficient to produce membrane ruptures of several micrometers long and, at intermediate intensities of 6–7 TW/cm^2^, to sever single-cell isthmuses in a single exposure.

Taken together, these findings imply that although very high temperatures and pressures exist transiently inside the submicrometer–micrometer plasma volume, the spatial extent of biologically relevant thermo-mechanical effects is limited to a radius of a few to ten micrometers around the beam focus. This situation contrasts with that of clinical millisecond diode lasers operating at 1480 nm, where the much longer pulse duration (typically 0.6 ms or more) allows heat to diffuse laterally during the pulse itself. Numerical modeling of such systems [14,28] has predicted peak temperatures of several hundred degrees at the beam center, as well as ellipsoidal isotherms extending well beyond the geometric beam focus, over distances ranging from several to tens of micrometers. In practical terms, femtosecond pulses concentrate energy deposition within a much smaller focal volume and on a time scale far shorter than the characteristic time of thermal diffusion, so that most of the excess energy is released mechanically via cavitation and shock waves rather than as sustained heating of the surrounding trophectoderm cells. This physical distinction provides a mechanistic rationale for the absence of visible thermal damage, no or moderate biological effects, and the preservation of implantation rates observed in the present study discussed below.

The safety of trophectoderm biopsy using femtosecond laser pulses of the indicated intensity was assessed using two groups of indicators: molecular markers of cellular stress (ROS and HSP70) and long-term reproductive outcomes (implantation and pregnancy outcome structure). Analysis of oxidative stress markers revealed that ROS levels in the E1 and E2 experimental groups after biopsy did not differ statistically from the negative control “C−” group. The Hodges–Lehmann estimates for the E1–“C−” and E2–“C−” differences are close to zero, and their 95% confidence intervals cross zero, indicating an absence of a significant ROS increase relative to the physiological baseline. Conversely, a pronounced increase in HSP70 relative to the “C−” group was observed (HL estimates are substantially greater than zero, and Cliff’s delta is close to one), indicating the activation of heat-shock proteins as part of the protective stress response rather than direct cytotoxic damage. Notably, this molecular evidence of an adaptive rather than damaging response is consistent with the absence of visible morphological or cytotoxic anomalies at the relevant time scales. No membrane blebbing or cytoplasmic opacification was observed during the brief laser exposures (seconds to tens of seconds; see Section 2.2). Several hours later, once the blastocyst had re-expanded (see Section 2.4), no anomalies or PI-positive (dead) cells were detected, except for a single isolated case.

It should be emphasized that the dose-dependent increase in HSP70 with intensity does not indicate an adverse effect. Since HSP70 induction was highest at an intensity of 16 TW/cm^2^ without a corresponding rise in ROS, this exposure regime was deliberately selected as the worst-case condition to evaluate implantation and developmental outcomes at the whole-organism level (Section 2.6). The obtained implantation data did not reveal a statistically significant negative effect of femtosecond laser trophectoderm biopsy on the overall implantation rate when the maximum intensity of 16 TW/cm^2^ was used. The risk difference for total implantation between the experimental and control groups was −3.80 percentage points. The 95% CI (−12.41 to +4.81 percentage points) does not indicate a significant decrease in implantation frequency but is compatible with a worsening of implantation of up to approximately 12 percentage points. Likewise, the relative risk and odds ratio of implantation (RR ≈ 0.87; OR ≈ 0.83; 95% CIs include 1) do not confirm a deterioration in outcomes. For the individual outcome categories (E, UE, and R), the wide confidence intervals reflect limited power for rare events and do not allow for the exclusion of small effects. The study’s limitations include small sample sizes in individual outcome categories and variability in implantation across series.

The absence of a statistically significant impairment of implantation at an intensity of 16 TW/cm^2^ therefore provides direct functional confirmation that the observed cellular stress response, although the most pronounced among the tested conditions, is compatible with normal reproductive outcomes. Consequently, 16 TW/cm^2^ can be regarded not only as the most efficient exposure condition in terms of pulse number, but also as the condition for which biological safety has been most directly validated, since it corresponds to the highest cellular stress load of those examined.

Direct quantitative comparison between the present femtosecond dataset and clinical millisecond systems is limited by differences in species, wavelength, pulse duration, reported exposure metrics, biopsy geometry, and outcome definitions. Published clinical data nevertheless provide a useful practical benchmark. Vitrolife (Octax Laser Shot/NaviLase) [29] and Eppendorf [30] protocols indicate that 2–3 laser pulses are usually sufficient for trophectoderm biopsy after stretching cell junctions, while the “normal” range is fewer than five shots per biopsy. Document [29] does not specify the exact energy of the millisecond laser pulses but notes that it should be 2–3 times higher than that required for *zona pellucida* dissection.

A study using the Lykos laser (Hamilton-Thorne) [31] showed that, in practice, the number of pulses can range from 2 to 21, with a median of four pulses (interquartile range 3–5, 95th percentile of 8). Increasing the number of pulses to these values does not result in poorer DNA amplification quality or worse clinical outcomes of PGT cycles in humans. Thus, the “working” range for clinical millisecond laser systems is approximately 3–5 pulses, with an acceptable “tail” of up to 7–8 pulses. However, according to the authors’ statement, a gold standard for TE biopsy has yet to be developed. Against this background, our results for femtosecond laser pulses with an intensity of 12–16 TW/cm^2^ show that at typical isthmus widths of 10–20 µm, the expected mean number of pulses required for biopsy sample separation is comparable to or lower than that in clinical millisecond protocols. This is observed in the absence of signs of an increase in reactive oxygen species, with a limited effect on implantation (according to RD/RR/OR with confidence intervals) and an adaptive nature of the HSP70 response.

These results indicate that the proposed femtosecond mode is at least as technologically efficient as clinical millisecond systems in terms of pulse number. However, this efficiency comparison does not by itself establish an equivalent biological comparison with millisecond TE biopsy, for which there is currently no comparable cellular stress-marker data. When assessing the safety of clinical millisecond lasers for embryo biopsy, the literature has focused primarily on functional and clinical criteria rather than molecular markers. These criteria include the ability of embryos to develop into blastocysts, implant, and produce live births of healthy offspring, as well as the stability of genetic testing results. Clinical studies summarized in [31] have demonstrated that, during human TE biopsy using 2–5 (sometimes up to 7–8) pulses, there is no observed decrease in implantation or live birth rates, deterioration in neonatal outcomes, or increase in mosaicism or non-informative genetic results.

Regarding cellular stress markers, the only relevant study on mice [32] examined *zona pellucida* drilling (assisted hatching) with a millisecond laser and found no induction of *Hsp70i* transcription. However, this procedure differs mechanistically from TE biopsy and cannot be treated as an equivalent biological comparator. To our knowledge, no published study has reported ROS or HSP70 measurements specifically for millisecond TE biopsy. Therefore, a matched molecular-level comparison with the femtosecond protocol used here is currently not possible from the literature. The present study therefore provides, to our knowledge, the first direct cellular stress-marker data (ROS, HSP70) for TE biopsy with any laser modality. This establishes a baseline against which future millisecond comparisons and refinements of the femtosecond protocol can be evaluated.

Even in the absence of a matched millisecond-laser control for TE biopsy, the significance of the present study lies in establishing an integrated quantitative framework for femtosecond TE biopsy, linking isthmus geometry and pulse intensity to biopsy efficiency, cellular stress responses, and implantation outcomes within a single experimental model. This framework defines a biologically tested operating window in which femtosecond biopsy is technically efficient and compatible with preserved implantation, thereby providing a rational basis for future head-to-head comparisons with clinical millisecond protocols rather than implying superiority in the absence of such a comparison. Together with our previous demonstration of femtosecond laser-assisted hatching on the same experimental platform, these findings further indicate that femtosecond irradiation can support both *zona pellucida* opening and trophectoderm separation within a single microsurgical modality. Importantly, in our previous direct comparison of assisted-hatching modalities, blastocyst collapse was observed after millisecond-laser treatment but not after femtosecond zona opening [18], suggesting an additional practical advantage of femtosecond irradiation during this stage of embryo micromanipulation.

### Study Limitations and Future Directions

When interpreting the results, it is important to consider the limitations of this study. These include the limited sample sizes in the experiments on biological effects and in the study of the implantation rate. The chi-squared test of the implantation-outcome distribution (E, UE, R) should be interpreted with caution owing to small expected frequencies (less than five) in the UE category, as noted above. Additionally, the study presents data only on two biological markers (ROS and *Hsp70*), which limits the generalizability of conclusions about the safety of laser exposure at the considered intensities. These limitations require caution when transferring results to other models and clinical protocols, and justify subsequent studies with increased sample sizes and refinement of the safe exposure parameter range. Experiments involving biopsy and subsequent development to live birth of mice would also be of interest to assess the overall health and viability of the fetuses.

A key limitation of clinical interpretation is the lack of a direct, matched comparison with a TE biopsy performed using a millisecond laser. Such a comparison would necessitate a distinct animal experiment involving harmonized biopsy geometry, exposure endpoints, biological assays and embryo-transfer outcomes. Consequently, the present manuscript is limited to assessing the feasibility and safety profile of the tested femtosecond protocol, and does not claim to be superior to clinical millisecond systems in an experimental context. However, such a comparison would be useful in the future to demonstrate the respective advantages and disadvantages of different solutions.

The physical interpretation of the femtosecond optical breakdown regime presented in this study is qualitative rather than quantitative: we did not perform dedicated numerical modeling of cavitation bubble formation, nor did we calculate the resulting temperatures and pressures for our specific exposure parameters (514 nm, 280 fs, 5–16 TW/cm^2^). The estimates of spatial and thermal localization discussed above rely on analogy with published data obtained under similar laser intensities. A parameter-specific simulation would be required to obtain quantitative values applicable directly to our experimental conditions.

Our ROS and HSP70 assays did not include unstained (no-dye) or no-primary-antibody controls, and therefore do not directly quantify the contribution of endogenous embryo autofluorescence to the measured fluorescence signal. Because all groups were processed with an identical staining protocol, autofluorescence is expected to contribute similarly across groups as a stable background component, limiting its impact on between-group comparisons. However, we cannot exclude that oxidative or thermal stress conditions used to generate the positive controls, or the laser exposure itself, altered the intrinsic autofluorescence of embryonic tissue. Future studies should include unstained and no-primary-antibody controls to formally quantify this contribution.

These studies were conducted on house mouse embryos. It is necessary to validate the conclusions drawn from the current study model with respect to optimal laser exposure parameters and the number of laser pulses required for dissection before they can be transferred directly to humans.

## 4. Materials and Methods

### 4.1. Animals

Outbred CD1 (ICR) mice in the natural reproductive cycle were used in the experiments. To obtain embryos, females and males of this line were mated under natural conditions. On the morning of the day following co-housing of animals of opposite sex in one cage, the presence of a vaginal plug (a marker of mating) was checked. If a vaginal plug was present, the females were separated and the day of plug detection was taken as E0.5, where E denotes the day of embryonic development.

The mice were maintained and cared for in a conventional vivarium. The mice received water and feed *ad libitum*. The animals were kept at a temperature between 22 and 24 °C under a 12 h light cycle. Mice were transported from the vivarium at IGB RAS by road vehicle to the laboratory of laser impact at JIHT RAS at stages E0.5–E2.5, where they were maintained under similar controlled conditions. Transportation took no more than 90 min. Pseudopregnant females also participated in the experiments. These females were obtained by mating with vasectomized males. The pseudopregnant females served as surrogate mothers for transferring manipulated and control embryos.

### 4.2. Embryo Collection and Manipulation

At stage E3.5, the fertilized females were sacrificed by cervical dislocation. Their uteri were then isolated and flushed with an M2 solution to search for embryos. The embryos were then placed in four-well culture plates containing pre-warmed, gassed EmbryoMax medium (Merck, Darmstadt, Germany). The embryos were cultured under standard incubator conditions (Galaxy 48S, New Brunswick Scientific, St Albans, UK) at 37 °C and 5% CO_2_.

### 4.3. Assessment of Reactive Oxygen Species Level

To analyze the presence of reactive oxygen species in embryos after laser exposure, the latter were stained with CM-H2DCFDA (Invitrogen, Thermo Fisher Scientific, Carlsbad, CA, USA, cat. # C6827) according to the manufacturer’s instructions. Briefly, the embryos were incubated in 1 µM CM-H2DCFDA diluted in KSOM medium (EmbryoMax) for 30 min in a CO_2_ incubator at 37 °C and 5% CO_2_. Negative and positive control groups were included in the study. The negative control group consisted of embryos that were maintained in a CO_2_ incubator (37 °C, 5% CO_2_) and were not subjected to laser exposure. The positive control group consisted of embryos that were incubated in 500 nM H_2_O_2_ diluted in EmbryoMax culture medium for 30 min in a CO_2_ incubator to stimulate ROS formation. The embryos were then washed three times for five minutes in HEPES-containing medium M2 to remove the peroxide before further manipulation. After staining, the embryos were also washed three times for five minutes each in M2 to remove the staining solution. To identify non-viable cells, additional staining was performed with the fluorescent dye propidium iodide (PI, Thermo Scientific, Waltham, MA, USA) at a working concentration of 500 nM.

### 4.4. Assessment of Heat-Shock Protein Expression Level

To analyze the presence of heat-shock proteins in embryos after laser exposure, the latter were first stained with primary antibodies against the mouse HSP70 protein (Cell Signaling, Danvers, MA, USA) and then with secondary antibodies against rabbit immunoglobulin G (IgG) conjugated to the fluorescent dye Alexa Fluor 488 (Cell Signaling), according to the manufacturer’s recommendations. The embryos were fixed with 4% paraformaldehyde (PFA) and permeabilized with 0.3% Triton X-100 (Suzhou Yacoo Science Co., Ltd., Suzhou, China) in PBS (PanEco). To block non-specific antibody binding, the embryos were placed in a solution containing 5% fetal bovine serum (FBS) (Cytiva) and 0.1% Triton X-100 in PBS. The primary antibodies were diluted at a ratio of 1:50 in a solution of 1% BSA (bovine serum albumin) in PBS buffer, and the secondary antibodies were diluted at a ratio of 1:500. After blocking, the embryos were incubated with the primary antibodies overnight at 4 °C, followed by a one-hour incubation with the secondary antibodies at room temperature. Nuclei were visualized using the fluorescent DNA stain DAPI (4′,6-diamidino-2-phenylindole) (Wuhan Servicebio Technology Co., Ltd., Wuhan, China).

The experimental embryo group (after femtosecond laser biopsy) was compared with the negative and positive control groups (five to ten embryos in each group). The negative control group was cultured in a CO_2_ incubator (37 °C and 5% CO_2_). The positive control group embryos were incubated in EmbryoMax culture medium in a CO_2_ incubator at a higher temperature (43 °C, 5% CO_2_) for three hours to stimulate HSP formation. The embryos were then fixed in PFA. After staining, the embryos were washed three times for five minutes in PBS to remove the staining solution.

After staining, the embryos were transferred to glass-bottomed Petri dishes containing drops of PBS covered with mineral oil (liquid paraffin, Origio a/s, Måløv, Denmark) to prevent evaporation and examined using a Leica STELLARIS 5 confocal microscope (Leica Microsystems GmbH, Wetzlar, Germany).

### 4.5. TE Cell Biopsy

For the biopsy, the embryos were placed in specialized glass-bottom Petri dishes containing two 20 µL drops of M2 medium. No more than three embryos were placed into each drop during a single procedure. The drops were then covered with mineral oil. The embryos in the first drop underwent the biopsy procedure, while the embryos in the second drop served as a parallel control. The procedure took no more than 5 min. During this time, TE cells, which play a role in implantation but are not included in the structures of the future newborn, were biopsied. The biopsy sample contained approximately 5 cells, which corresponds to the standard practice used in IVF (in vitro fertilization) clinics [33].

In a previous study [21], it was shown that biopsy using single femtosecond pulses is more efficient than the pulsed-periodic exposure mode. This method is also used in clinics with millisecond laser dissectors. We also selected this mode, in which a single femtosecond laser pulse of a specified intensity is delivered to the point indicated by the operator on the blastocyst image on the personal computer screen.

### 4.6. Laser-Assisted Hatching

In the absence of signs of natural hatching at the blastocyst stage, LAH was performed prior to the biopsy procedure. After orienting the embryo in the Petri dish with micropipettes, it was held with a holding pipette on the side of the inner cell mass. The operator applied the laser incision trajectory to the embryo image using specialized software. LAH was performed using a sequence of femtosecond laser pulses with an intensity of 2.5 TW/cm^2^ delivered at a repetition rate of 2.5 kHz along a preset laser-beam trajectory at a speed of 100 µm/s. This resulted in either a complete opening of the ZP or sufficient thinning for the biopsy pipette to penetrate (see Figure 1).

### 4.7. Embryo Selection for Transfer

A total of 836 E3.5-stage embryos were obtained during the study. For the TE biopsy procedure, it is necessary that the embryo reach the expanded blastocyst stage during its development. Biopsies were performed only on embryos that had not completed the hatching process (i.e., the emergence of the embryo from its ZP). This natural process occurs before implantation. Hatching could also occur at night when biopsies could not be performed. Embryos that had undergone the hatching stage and left the *zona pellucida* were not used in the experiments. Thus, a total of 417 embryos (221 in the experimental group and 196 in the control group) were transferred to pseudopregnant females for implantation experiments after biopsy.

### 4.8. Embryo Transfer

Embryo transfer was performed after the biopsy procedure at developmental stage E3.5 or E4.5 if delayed blastocyst development was observed. The procedure was carried out on a “day-to-day” basis, with the duration of mouse pseudopregnancy corresponding to the developmental age of the embryo. The transfer was performed according to the standard protocol. After anesthetizing the animal and treating the surgical site with 70% alcohol, the skin and peritoneum were incised. The ovary–oviduct–uterus complex was exteriorized and fixed with clamps. Then, the uterine wall was perforated with an insulin syringe needle, and a glass capillary containing the culture medium with the embryos was inserted into the opening. The embryos were expelled from the capillary into the uterine horn lumen using a mouth pipette. Then, the ovary–oviduct–uterus complex was returned to the abdominal cavity. The peritoneum and skin were sutured layer by layer. Finally, the animal was placed in a clean cage.

Since the mouse had a bicornuate uterus, embryos from both the control and experimental groups were transferred into it. The experimental embryos were transferred into the left horn and the control embryos in the right. This made it possible to exclude the influence of the maternal factor on the success of transplantation and implantation. The number of embryos transferred to each horn was 6–10.

### 4.9. Femtosecond Laser Scalpel

**Figure 10:**
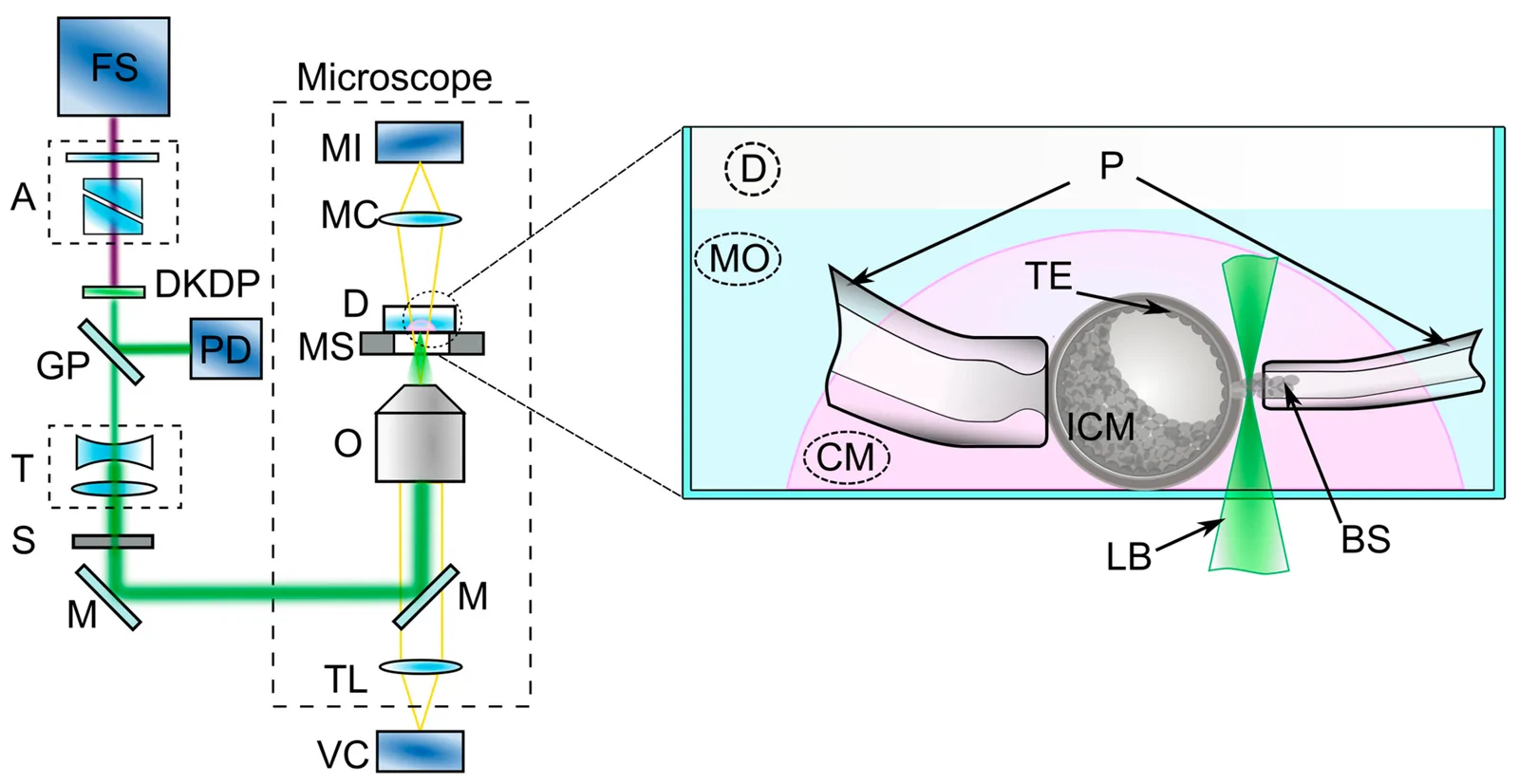
Experimental setup. FS—femtosecond laser source; A—attenuation unit; DKDP—DKDP crystal; GP—glass plate; PD—photodiode; T—telescope; S—mechanical shutter; M—mirror; O—20× objective; MS—motorized stage; D—Petri dish with embryo; MC—microscope condenser; MI—microscope illuminator; TL—tube lens; VC—video camera; MO—mineral oil; CM—culture medium droplet; ICM—inner cell mass; TE—trophectoderm; BS—biopsy sample; P—pipettes; LB—laser beam.

A trophectoderm biopsy was performed using a custom-built femtosecond laser microsurgery platform (Figure 10) that was integrated with an inverted microscope (IX-71, Olympus, Minnetonka, MN, USA). The system was based on a femtosecond ytterbium laser, which delivered 280 fs pulses at a repetition rate of 2.5 kHz. In the present study, second-harmonic radiation at 514 nm from a nonlinear DKDP (deuterated potassium dihydrogen phosphate) crystal was used for embryo microsurgery. The laser beam was introduced into the side port of the inverted microscope via an attenuation unit and a telescope, and was then focused using a 20× lens (UPLFLN, Olympus) with a numerical aperture of 0.5. This provided a focal spot radius of the laser beam of approximately 1.4 μm at the 1/e level.

The energy of the laser pulses was controlled using a polarization-based attenuator and monitored using a photodiode (DET36A2, Thorlabs, Inc., Newton, NJ, USA), which received a small portion of the reflected beam from a thin glass plate. The photodiode signal was digitized using an oscilloscope (TDS5054, Tektronix) and calibrated using a power meter consisting of a photodiode sensor (S120VC, Thorlabs, Inc., Newton, NJ, USA) and console (PM100D, Thorlabs, Inc., Newton, NJ, USA). During calibration, the sensor was placed temporarily on the motorized microscope stage (SCAN IM 120 × 80, Märzhäuser, Wetzlar GmbH & Co. KG, Wetzlar, Germany) in order to measure the radiation transmitted through the lens.

The microscope was equipped with a micromanipulation module with biopsy pipettes, which were used for blastocyst positioning and trophectoderm isolation. During the procedure, two pipettes mounted in dedicated holders and coupled to three-axis oil-hydraulic micromanipulators (Narishige Scientific Instrument Lab., Tokyo, Japan) were used. A video camera (DFK 72AUC02, The Imaging Source Europe GmbH, Bremen, Germany) provided real-time visualization on the monitor of a personal computer. The procedure and irradiation mode were controlled using custom-built LabVIEW 2019 software (National Instruments Corporation, Austin, TX, USA), including single-pulse mode for biopsy and multi-pulse (series of pulses at a repetition rate of 2.5 kHz) mode for LAH.

### 4.10. Statistical Analysis

The Shapiro–Wilk test was used to evaluate the normality hypothesis. A statistical analysis was performed using the Mann–Whitney U test with Bonferroni correction for multiple comparisons. For the analysis of categorical variables, contingency tables were constructed, and chi-square tests were calculated. In addition to *p* values, effect sizes were calculated: the Hodges–Lehmann estimate (median difference between groups) and Cliff’s delta (a probabilistic measure of the superiority of one group over another), as well as 95% confidence intervals for these estimates [34,35]. These calculations made it possible to quantitatively describe not only the presence or absence of statistical significance but also the magnitude of the effect and the degree of overlap between group distributions. The Hodges–Lehmann estimate and Cliff’s delta were calculated in the Python 3.13 environment using the NumPy libraries, and the 95% confidence intervals were obtained using a nonparametric bootstrap method.

The *N*(*D*) relationship was modeled by linear regression, including a model with a *D* × *I* interaction term whose significance was assessed with a multivariate Wald test; 95% confidence and prediction bands were constructed for the dataset obtained for intensity of 16 TW/cm^2^. Significance of regression coefficients was evaluated by robust *t*-tests using heteroscedasticity-consistent (HC3) standard errors.

For the risk difference (RD), as well as for the relative risk (RR) and the odds ratio (OR), 95% confidence intervals were calculated. These intervals allowed us to quantify the maximum possible improvement or worsening in implantation.

### 4.11. Corrected Total Cell Fluorescence

Fluorescence intensity was quantified in ImageJ (version 1.52e). For each embryo, confocal Z-stacks were split by fluorescence channel, and sum projections were generated for the ROS and HSP70 channels. The embryo region of interest (ROI) was segmented on the sum projection using the Huang automatic threshold. The ROI area (*A*), integrated density (IntDen), and mean gray value of the background (BG_mean_) were then measured.

A two-dimensional corrected total cell fluorescence was first calculated as CTCF_2D_ = IntDen − (*A* × BG_mean_). Since sum projections combine signal from all optical sections, the integrated density also depends on the number of Z-slices acquired for each embryo. To account for differences in embryo size and stack depth, CTCF_2D_ was normalized to the embryo volume, *V* = *A* × *n* × Δ*z*, where *n* is the number of optical slices and Δ*z* = 2 µm is the Z-step used in our confocal acquisition. The volume-normalized value, CTCF = CTCF_2D_/*V*, was used as the fluorescence metric for all subsequent statistical comparisons of ROS and HSP70 levels between groups.

## 5. Conclusions

The study showed that femtosecond laser biopsy of mouse blastocyst trophectoderm cells at intensities of 12–16 TW/cm^2^ allows for a balance of technological efficiency and an acceptable safety profile. The linear dependence of the number of pulses on isthmus width indicates the practical feasibility of such biopsies under conditions of limited manipulation time and minimized total energy input. Most procedures are performed in 2–4 pulses, even for relatively wide isthmuses (on the order of 20–30 µm). The absence of an increase in ROS levels, compared to the negative control, along with limited HSP70 expression elevation, and the preservation of the implantation rate and pregnancy outcome structure at the level of the control group, indicates that the considered femtosecond mode does not cause significant thermal or lethal damage. Rather, it mainly triggers an adaptive stress response that does not result in a noticeable deterioration of reproductive outcomes in the model used. Within the 12–16 TW/cm^2^ range, 16 TW/cm^2^ offers the best overall balance between efficiency and safety: it minimizes the number of pulses and total exposure time for wide isthmuses, does not elevate ROS, and was the specific condition for which the absence of a statistically significant worsening of implantation was directly confirmed at the whole-organism level. The dose-dependent rise in HSP70 with intensity therefore reflects a proportionate, adaptive response rather than a safety concern within the tested range.

Taken together, these results establish a quantitative and biologically validated operating framework for femtosecond TE biopsy. This framework provides a basis for further optimization of the technique and for rigorous head-to-head comparison with millisecond laser protocols under matched experimental conditions.

## Figures and Tables

**Table 1:** Statistical analysis of ROS values; Mann–Whitney U test, *p* values.

ROS	C−	*I* = 12 TW/cm^2^	*I* = 16 TW/cm^2^	C+
**C−**	–	0.09524	0.90476	0.00794
***I* = 12 TW/cm^2^**	0.09524	–	0.06349	0.00794
***I* = 16 TW/cm^2^**	0.90476	0.06349	–	0.01587
**C+**	0.00794	0.00794	0.01587	–

**Table 2:** Statistical analysis of *Hsp70* values, Mann–Whitney U test, *p* values.

*Hsp70*	C−	*I* = 12 TW/cm^2^	*I* = 16 TW/cm^2^	C+
**C−**	–	0.00032	0.00032	0.00046
***I* = 12 TW/cm^2^**	0.00032	–	0.15079	0.00794
***I* = 16 TW/cm^2^**	0.00032	0.15079	–	0.00794
**C+**	0.00046	0.00794	0.00794	–

**Table 3:** Number of implanted embryos per uterine horn.

	Experiment			Control		
	E	UE	R	E	UE	R
*N*_i_	26	3	28	30	3	25
*K*_ir_ , %	11.76	1.36	12.67	15.3	1.53	12.76

## Data Availability

The original contributions presented in this study are included in the article. Further inquiries can be directed to the corresponding author.

## Abbreviations

The following abbreviations are used in this manuscript:

BSA	Bovine serum albumin
DAPI	4′,6-diamidino-2-phenylindole
DKDP	deuterated potassium dihydrogen phosphate
FBS	Fetal bovine serum
Fs	Femtosecond
HSP	Heat-shock proteins
ICM	Inner cell mass
IVF	In vitro fertilization
LAH	Laser-assisted hatching
ms	Millisecond
OR	Odds ratio
PBS	Phosphate-buffered saline
PFA	Paraformaldehyde
PGT	Preimplantation genetic testing
PI	Propidium iodide
RD	Risk difference
ROS	Reactive oxygen species
RR	Relative risk
TE	Trophectoderm
ZP	*Zona pellucida*
